# Effects of proprioceptive stimulation foot pads on in-toeing gait in children: a retrospective study

**DOI:** 10.1186/s13018-025-06644-9

**Published:** 2026-02-01

**Authors:** Yulong Ben, Jing Chen, Danfeng Zheng, Ying Chen, Pengfei Zheng

**Affiliations:** 1 Department of Orthopaedic surgery, Children’s Hospital of Nanjing Medical University, Nanjing, 210000 Jiangsu Province China; 2 National Health Commission Contraceptives Adverse Reaction Surveillance Center, Nanjing, 210000 Jiangsu Province China; 3 Jiangsu Provincial Medical Key Laboratory of Fertility Protection and Health Technology Assessment, Nanjing, 210000 Jiangsu Province China; 4 Jiangsu Health Development Research Center, Nanjing, 210000 Jiangsu Province China

**Keywords:** Proprioceptive stimulation foot pads, In-toeing gait, Children, Gait analysis

## Abstract

**Background:**

In-toeing gait is a common developmental condition in children and may lead to gait instability, frequent falls, and discomfort. However, evidence supporting conservative interventions, such as proprioceptive stimulation foot pads, remains limited.

**Objective:**

To evaluate the effects of proprioceptive stimulation foot pads on gait parameters in children with in-toeing gait.

**Methods:**

This retrospective study included 119 children aged 5–12 years who were diagnosed with in-toeing gait in Children’s Hospital of Nanjing Medical University between January 2020 and April 2023. Based on clinical records, children who received proprioceptive stimulation foot pads were assigned to the treatment group, while those managed by observation alone comprised the control group. Gait parameters measured at baseline and follow-up were extracted for analysis.

**Results:**

Compared with the control group, the treatment group demonstrated a significant improvement in foot progression angle. In contrast, walking speed, step length, stride length, arch index, and plantar pressure showed changes in both groups, with no consistent between-group differences.

**Conclusion:**

Proprioceptive stimulation foot pads are associated with improved foot progression angle in children with in-toeing gait, while effects on other gait parameters appear limited. These findings provide preliminary support for the use of this conservative intervention.

**Supplementary Information:**

The online version contains supplementary material available at 10.1186/s13018-025-06644-9.

## Introduction

In-toeing gait is a common concern in pediatric orthopedic practice [1] and is typically attributed to increased femoral anteversion, internal tibial torsion, or metatarsus adductus [2]. Children with in-toeing gait may present with gait instability, frequent tripping, and fatigue-related discomfort [3]. These symptoms often increase parental anxiety and may interfere with daily functioning. Although most cases improve spontaneously during early childhood as musculoskeletal structures mature [4–6], a subset of children continue to demonstrate an abnormal increased foot progression angle (FPA) beyond the expected age of correction.

There is no consensus regarding the optimal timing or strategy for intervention. Some authors recommend deferring treatment until late childhood [7], whereas others have reported that persistent in-toeing gait after approximately six years of age is associated with residual deformity and future biomechanical problems [8, 9]. Prolonged abnormal gait patterns may contribute to compensatory lower limb malalignment, increased risk of hip and patellofemoral pathology, and psychosocial concerns, underscoring the need for effective conservative management options [10, 11].

It is generally believed that children diagnosed with in-toeing gait at five or six years of age who show no signs of improvement after two years of observation should receive active treatment [12]. A variety of non-surgical interventions have been explored, including physical therapy, gait training, and orthotic devices. Conventional orthoses, such as rotation braces, can be effective [13] but are frequently limited by poor compliance and interference with daily activities. As a more child-friendly alternative, proprioceptive stimulation foot pads have gained increasing attention for their potential to influence neuromotor control through targeted plantar sensory input.

Proprioceptive stimulation foot pads provide plantar sensory input through thin, in-shoe wedges, thereby modulating neuromuscular control and postural alignment [14–19]. Despite their growing clinical use, evidence supporting their efficacy in children with in-toeing gait—particularly in Chinese pediatric populations—remains limited.

Given these gaps in the literature and the clinical demand for conservative, well-tolerated treatment options, further investigation into the role of proprioceptive stimulation foot pads in correcting in-toeing gait is warranted. Therefore, this study aimed to evaluate the effects of proprioceptive stimulation foot pads on gait parameters in children with persistent in-toeing gait using a retrospective cohort design. By analyzing objective gait measurements before and after treatment, this study seeks to provide clinically relevant evidence to inform decision-making regarding the use of proprioceptive stimulation foot pads in pediatric gait correction.

## Methods

### Study design and setting

This study adopted a retrospective cohort design and was conducted in Children’s Hospital of Nanjing Medical University, a tertiary care center in Nanjing, China. Clinical data were retrospectively collected from the hospital’s electronic medical records database between January 2020 and April 2023.

Baseline and follow-up gait assessments were performed using the same standardized protocol. Follow-up evaluations were conducted at least 12 months after baseline for all participants. Gait data were extracted from the electronic records by an investigator who was not involved in the clinical decision to prescribe foot pads, and group allocation was masked during statistical analysis.

In our study, the key elements were described according to the PICOS framework:

#### Population (P)

Children aged 5–12 years who continued to exhibit in-toeing gait despite natural growth and a period of observation, representing a population requiring conservative management.

#### Intervention (I)

Proprioceptive stimulation foot pads are designed to stimulate plantar sensory receptors and improve postural alignment and motor control through neuromuscular feedback. Although previous studies have demonstrated that these devices can enhance balance and gait in general populations, their specific effects on in-toeing gait in children remain unclear.

#### Comparison (C)

To assess clinical effectiveness, the present study compared children who used proprioceptive stimulation foot pads with those who received observation alone.

#### Outcomes (O)

Outcome measures included changes in gait parameters—particularly FPA, walking speed, step length, and stride length—measured at baseline and follow-up, which provide objective indicators of functional improvement.

#### Study design (S)

A retrospective cohort design was used to evaluate these outcomes using clinical gait data extracted from hospital archives.

### Population and subgroups

This retrospective study reviewed the clinical data of 119 children diagnosed with in-toeing gait in Children’s Hospital of Nanjing Medical University between January 2020 and April 2023.

Participants were included if they were aged 5 to 12 years, had a clinical diagnosis of in-toeing gait (defined as a unilateral or bilateral FPA less than 3°), and demonstrated no significant improvement after at least two years of observation following diagnosis. In all cases, informed consent was obtained from the legal guardians.

Children were excluded [20, 21] if they were younger than 5 years or older than 12 years; had neurological disorders such as cerebral palsy or developmental abnormalities, including hip dysplasia, that could contribute to in-toeing gait; presented with concurrent skeletal, neurological, or metabolic disorders, or gait abnormalities resulting from trauma or surgery; or exhibited mixed etiologies (e.g., both femoral anteversion and tibial torsion). By focusing exclusively on children with isolated in-toeing gait, these criteria minimized heterogeneity and potential confounding factors in the analysis.

### Intervention and comparators

Based on medical records, children were categorized into treatment and control groups according to whether they received proprioceptive stimulation foot pads. The treatment group consisted of 100 children (53 boys and 47 girls) who were prescribed foot pads because of persistent in-toeing gait after a minimum of two years of observation, associated functional complaints (e.g., frequent tripping or parental concerns regarding gait appearance), and willingness to use orthotic insoles. The control group included 19 children (14 boys and 5 girls) who met the same diagnostic criteria but whose families opted for continued observation without orthotic intervention.

Gait data recorded at baseline and follow-up were analyzed for both groups, including FPA, walking speed, step length, stride length, arch index, and peak plantar pressure. Baseline comparisons between the treatment and control groups are presented in the Results section.

Although the group sizes were unequal, this disproportion reflects the clinical preference for prescribing proprioceptive stimulation foot pads during the study period, resulting in a higher number of treated cases. We acknowledge that this imbalance may affect statistical power and introduce potential bias; therefore, future studies with more balanced sample sizes are warranted.

### Outcomes and measurements

#### Outcomes

**Fig. 1:**
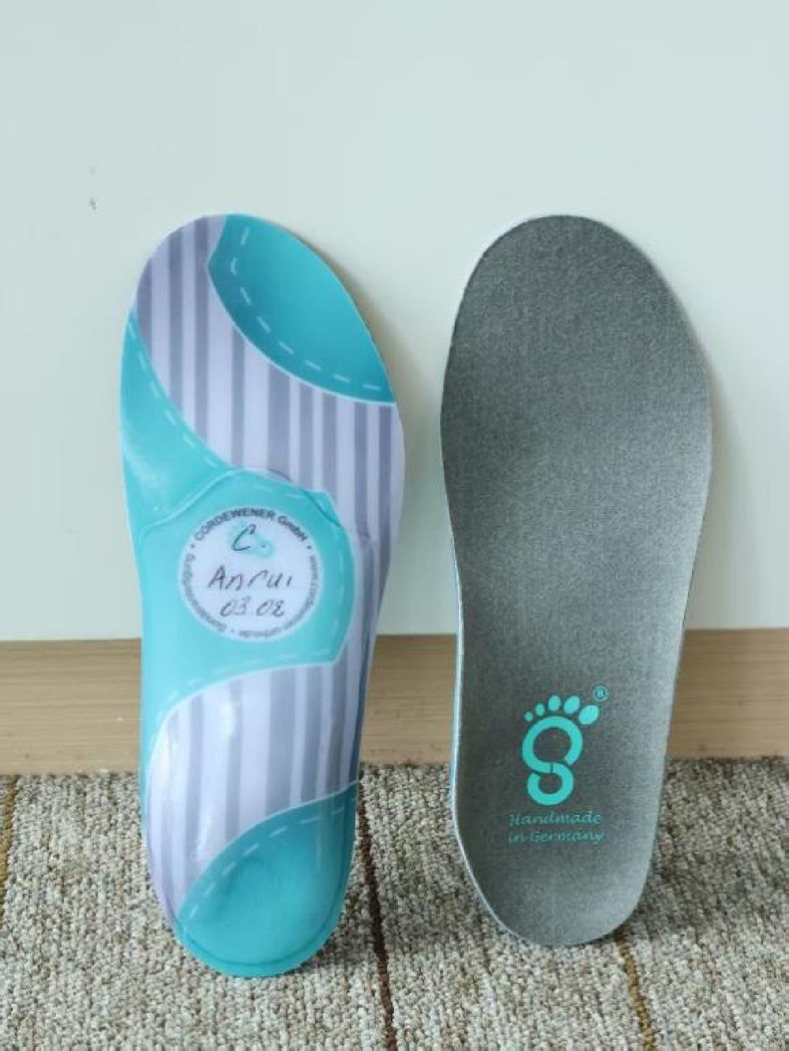
Illustration of the proprioceptive stimulation foot pad. Top view showing the soft, striped upper layer, highlighting its lightweight and child-friendly design. Bottom view displaying the gray silicone base with handmade craftsmanship ("Handmade in Germany") and foot imprint logo, illustrating the wedge-shaped elevations for plantar stimulation. These images demonstrate the foot pad's simple, non-electrical mechanism for sensory feedback in daily use

The primary gait parameters measured included FPA, walking speed, step length, stride length, arch index, and peak plantar pressure. Changes in these parameters were compared between the two groups at baseline and follow-up (Fig. 1).

FPA was measured dynamically during treadmill walking using the Zebris Gait Analysis System (version 2.0; Germany) and was defined as the angle between the longitudinal axis of the foot and the direction of progression. According to the sign convention, negative values indicate inward rotation (in-toeing; < 0°), positive values indicate outward rotation (external deviation; > 0°), and 0° represents neutral alignment. This parameter reflects lower limb rotational alignment. Normal pediatric values typically range from approximately − 5° to + 5°, and deviations beyond this range may contribute to gait instability in children with in-toeing gait.

The arch index was calculated as the ratio of the midfoot contact area to the total foot contact area during static barefoot scanning and was expressed as a percentage [(midfoot area/total foot area) × 100]. Values greater than 30% indicate pes planus (flatfoot). This parameter is clinically relevant in children with in-toeing gait, as flatfoot may contribute to lower limb instability and compensatory rotational patterns. Normal pediatric values generally range from approximately 20 to 30%. The arch index reflects longitudinal arch development and its role in gait stability.

#### Research foot pad

A specialized proprioceptive corrective foot pad designed for children with in-toeing gait was used as the intervention (Zebris; Germany). The device is a non-electrical, insole-like insert made of soft, flexible material that is placed inside the shoe beneath the plantar surface of the foot. Small wedges or pads are positioned beneath selected plantar regions (e.g., the heel, lateral forefoot, or medial arch) according to the neuromuscular modulation concept to provide localized mechanical stimulation to plantar sensory receptors (as illustrated in Fig. 1).

This mechanical sensory input is intended to modulate muscle tone and postural alignment through reflexive neuromuscular pathways, thereby reducing excessive internal rotation and promoting a more neutral foot progression pattern over time.

#### Research equipment

Gait analysis was performed using a treadmill-based system with integrated plantar pressure assessment (Zebris; Germany). This system is commonly used in clinical and commercial orthopedic settings and integrates treadmill walking, visual gait observation, and plantar pressure measurement.

Detailed manufacturer specifications of the plantar pressure measurement plate and the treadmill-based gait analysis and training system are provided in Supplementary Materials S1 and S2.

The cited studies [22, 23] supporting measurement reliability (Pedar-X and MatScan systems) were not intended as direct validation of the Zebris system itself, but rather as evidence that plantar pressure and spatiotemporal gait parameters can be measured reliably in pediatric populations using established in-shoe or pressure-mat technologies. Independent validation data for the Zebris system are currently unavailable; therefore, the results should be interpreted with appropriate caution.

#### Research procedure

According to archived clinical records, all children underwent a standardized two-stage assessment protocol to ensure comprehensive evaluation and exclusion of potential confounding factors.

##### Stage 1

Clinical history and physical examination.

Legal guardians were questioned regarding the child’s current physical condition, pain symptoms, family history of hereditary diseases, and daily physical activity habits. This was followed by a comprehensive physical examination, which assessed foot morphology (e.g., arch height and toe alignment) as well as overall lower limb and postural alignment, confirming a diagnosis of isolated in-toeing gait without neurological or skeletal comorbidities.

##### Stage 2

Instrumented gait analysis.

**Fig. 2:**
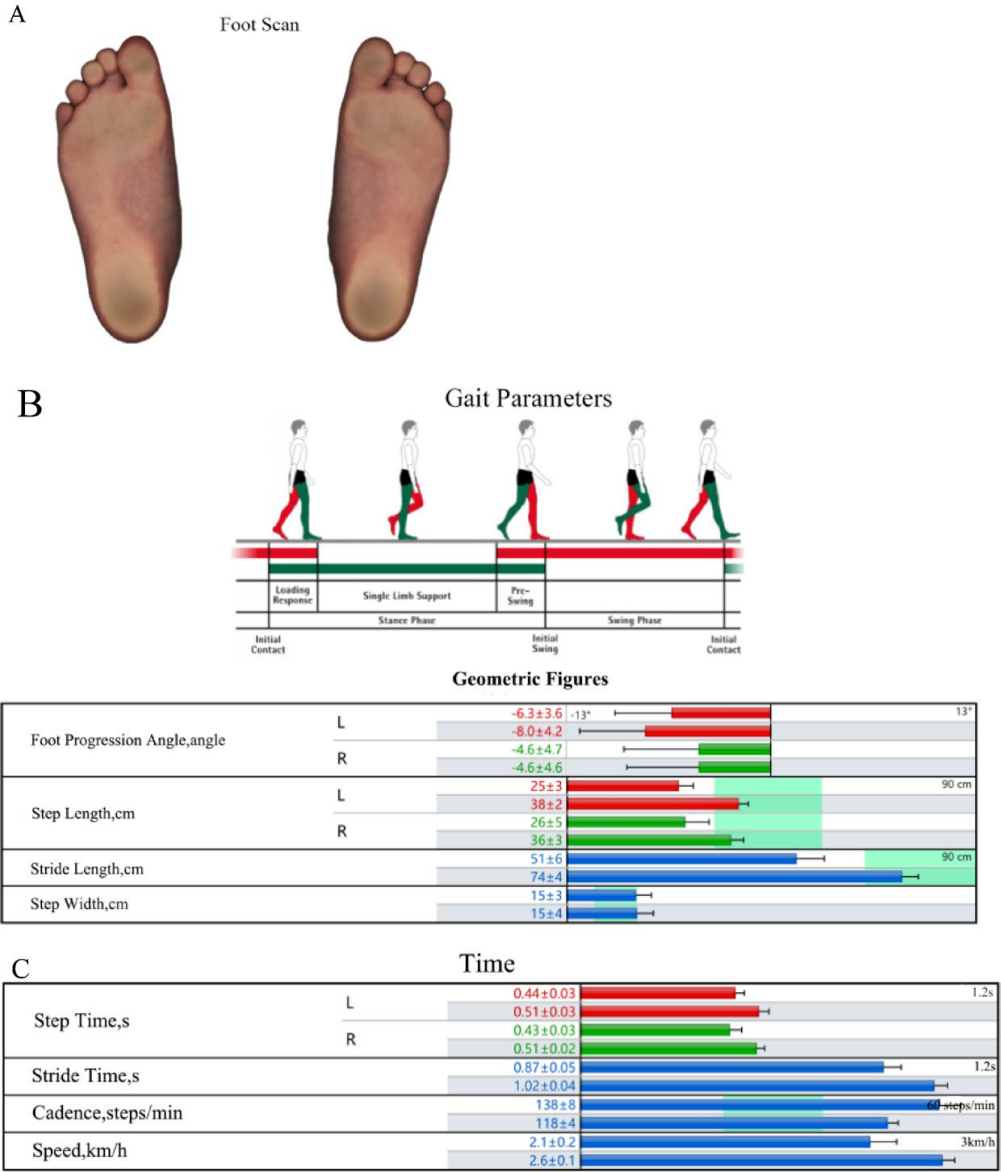
Typical workflow illustration of gait analysis using the Zebris system (Exemplary Case). **A** Foot Scan. Static foot scan displaying plantar regions and pressure areas (darker shades = higher pressure). **B** Gait Parameters. Geometric gait parameters (e.g., FPA, step/stride length). **C** Time Parameters. Temporal gait parameters (e.g., cadence, speed). Figure Legend Note: This figure illustrates the analysis process for a representative child (not group results). Solid elements = pre-intervention baseline; shaded = post-intervention follow-up. Detailed metrics in Sect. 2.4.1; advanced visuals in Supplementary Figure S3

Multidimensional gait data were collected using the Zebris system, including: (i) static barefoot foot scans to obtain baseline arch index and plantar pressure distribution (Fig. 2A); (ii) dynamic treadmill-based plantar pressure measurements to assess FPA, walking speed, and stride parameters (Fig. 2B and C); and (iii) synchronized high-definition video capture using the SYNCLightCam system for visual confirmation of gait phases (e.g., loading response to swing).

For dynamic assessments, treadmill speed was individualized to match each child’s natural overground walking pace (typically 2–3 km/h for children aged 5–12 years), thereby promoting a comfortable and non-fatigued walking condition. Each trial lasted approximately 30 s, and 4–5 repeated trials were performed to account for intra-individual variability. For analysis, the most representative dataset—defined as the trial closest to the child’s natural gait rhythm and yielding approximately 20–30 strides—was selected in accordance with the clinical protocol. Follow-up assessments (≥ 1 year after baseline) followed the same procedure, allowing for pre- and post-intervention comparisons based on medical records. Advanced dynamic visualizations (e.g., pressure trajectories) are provided in Supplementary Figure S1.

### Time horizon

Data were extracted from clinical records collected between January 2020 and April 2023, ensuring an adequate sample size and consistency in gait evaluation protocols.

### Analytical methods

Data analyses were performed using SPSS software (version 26.0). The normality of data distributions was assessed using the Shapiro–Wilk test.

For within-group comparisons of baseline and follow-up outcomes, paired t-tests were applied for normally distributed variables, whereas Wilcoxon signed-rank tests were used for variables that did not meet normality assumptions. Between-group comparisons were conducted using independent-samples t-tests for normally distributed data or Mann–Whitney U tests for non-normally distributed data.

Effect sizes were calculated to quantify the magnitude of intervention effects and to complement statistical significance testing. For parametric analyses, Cohen’s d was used to estimate effect size, with values of 0.2, 0.5, and ≥ 0.8 interpreted as small, medium, and large effects, respectively. When sample sizes were small, Hedges’ g was additionally reported as a bias-corrected estimate of effect size, applying the same interpretive thresholds.

For nonparametric analyses, effect sizes were calculated using the standardized test statistic (r), derived from the Z value divided by the square root of the total number of observations (r = Z/√N). Effect sizes were interpreted as small (0.1), medium (0.3), and large (≥ 0.5).

All statistical tests were two-tailed, and a p-value < 0.05 was considered statistically significant. Effect size estimates were used to aid interpretation of clinical relevance beyond statistical significance, with larger values indicating more substantial improvements in gait parameters, such as clinically meaningful correction of FPA in pediatric in-toeing.

### Characterizing heterogeneity and assumptions

Subgroup analyses were conducted based on age and baseline gait parameters to explore potential heterogeneity. The study assumed consistent data accuracy across clinical records. Given the retrospective nature of the study design, potential sources of bias—including selection bias, recall bias, and the lack of randomization—must be acknowledged. These inherent limitations may affect the internal validity of the results and limit the generalizability of the findings. Future studies employing prospective, randomized controlled designs are warranted to confirm these results.

Because this was a retrospective study, specific data on compliance with foot pad use could not be collected. As compliance is critical to the effectiveness of orthotic interventions, the absence of compliance data represents an important limitation of this study. Future prospective studies should incorporate compliance monitoring to evaluate its influence on treatment outcomes. In addition, variability in adherence to the intervention may have contributed to the observed effects on gait parameters; therefore, compliance should be carefully considered when interpreting the study results.

**Fig. 3:**
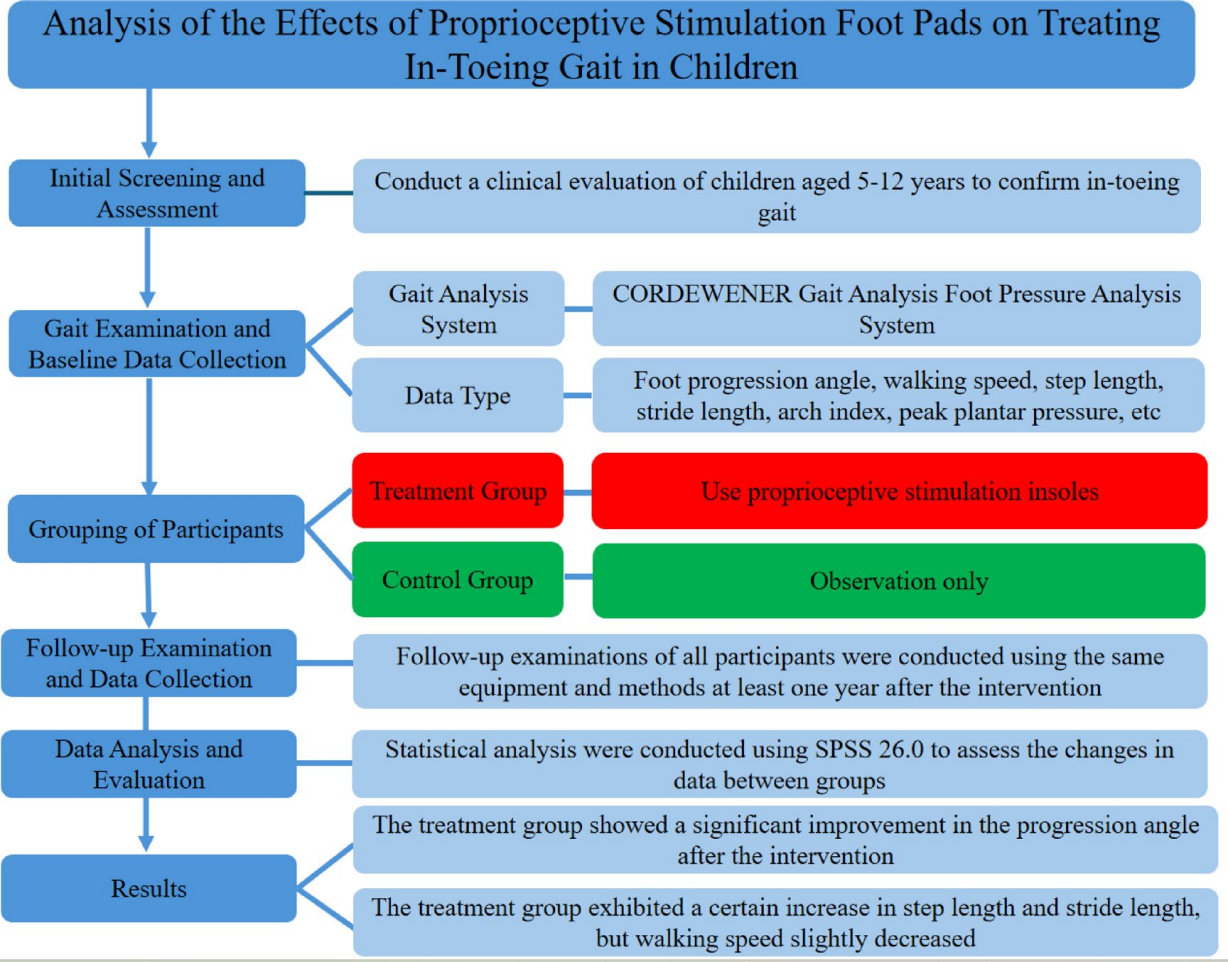
Flowchart of the study design. This flowchart summarized the study’s process for evaluating proprioceptive stimulation foot pads in children with in-toeing gait. Participants were screened and assessed at baseline, allocated to treatment (foot pads) or control (observation) groups based on records, followed up after ≥ 1 year, and analyzed using SPSS 26.0 for gait parameter changes

The overall study flow, including screening, eligibility assessment, group allocation, and data analysis, is illustrated in Fig. 3.

## Results

### Study parameters

A total of 119 children with in-toeing gait were included in the study (treatment group: 100; control group: 19). All participants completed more than one year of follow-up. Baseline demographic and clinical characteristics were comparable between the two groups (Table 1; *P* > 0.05). Gait parameters, including FPA, step length, stride length, walking speed, arch index, and peak plantar pressure, were recorded at baseline and at follow-up (Tables 2 and 3).

### Incremental outcomes

Within the treatment group, FPA improved significantly from baseline to follow-up for both feet, whereas no significant change was observed in the control group. Between-group comparisons at follow-up demonstrated significantly improved FPA in the treatment group compared with the control group for both feet, with large effect sizes. For spatiotemporal parameters (step length, stride length and walking speed), arch index, and plantar pressure measures, between-group differences at follow-up were not statistically significant. Detailed statistical results are presented in Tables 2 and 3.

Although incremental cost-effectiveness ratios were not directly calculated, the low-cost and non-invasive nature of the intervention suggests a potentially favorable cost-benefit profile compared with traditional orthotic or physiotherapeutic approaches. Future studies should incorporate formal economic evaluations to quantify cost-effectiveness.

### Characterizing uncertainty

Sensitivity analyses were conducted to assess the robustness of the results. Reanalysis excluding outliers (> 2 SD from the group mean) did not materially alter the statistical significance of the findings, confirming the stability of the observed effects. Standard deviations for gait parameters were moderate (4°–8° for FPA and 7–15 cm for stride length), indicating acceptable within-group variability. Treatment effects on FPA remained significant across all resampling iterations, reinforcing confidence in the intervention’s effect.

### Characterizing heterogeneity

Subgroup analyses revealed modest variations in treatment response according to age and baseline severity. Younger participants (< 8 years) tended to exhibit greater improvements in FPA than older children (*P* = 0.041), possibly reflecting greater plasticity in motor adaptation. No statistically significant sex-based differences were observed (*P* > 0.05). Greater variability in improvements in stride length and arch index was observed among participants with more severe baseline in-toeing, suggesting heterogeneity in treatment responsiveness related to the magnitude of the initial deformity.

### Summary of outcomes

Overall, the treatment group demonstrated a clear improvement in FPA following the intervention period, whereas no statistically significant change was observed in the control group. The proprioceptive stimulation foot pad group showed a significantly greater correction in FPA than the control group, indicating a clear between-group benefit in gait alignment.

In contrast, arch index values showed significant within-group improvement over time in both groups; however, between-group differences were neither consistent nor statistically significant.

Although walking speed decreased slightly at follow-up, this change may reflect compensatory gait adaptation during neuromotor retraining rather than functional decline. Taken together, these findings suggest that proprioceptive stimulation foot pads confer an additional benefit in correcting gait alignment, as reflected by improvements in FPA, whereas changes in arch index appear to be primarily time-related and do not demonstrate a clear added advantage over observation alone.

**Table 1:** Baseline data comparison between the two groups

Group	*n*	Gender (male/female)^a^	Age (years) ^b^	Height (cm) ^b^	Weight (kg) ^b^	BMI ^b^
Treatment	100	53/47	8.02 ± 1.92	130.88 ± 13.44	28.46 ± 9.33	16.27 ± 2.74
Control	19	14/5	7.82 ± 1.94	130.98 ± 15.55	29.02 ± 10.83	16.27 ± 2.45
Difference & 95% CI			0.20 (− 0.75 to 1.15)	− 0.10 (− 6.94 to 6.73)	− 0.56 (− 5.31 to 4.19)	− 0.00 (− 1.34 to 1.33)
*t-value or χ²*		2.777	0.419	− 0.030	− 2.234	− 0.004
*P-value*		0.096	0.676	0.976	0.815	0.997

^a^Chi-square test^b^t-test

**Table 2:** Within-Group changes in gait parameters at baseline and follow-up

Gait Parameters	Group	*n*	Baseline	Follow-up	*P*-value	effect sizes
Left foot progression angle (°)	Treatment	100	− 3.67 ± 4.46	− 0.24 ± 4.09	< 0.001	Cohen’s d = 0.861
	Control	19	− 5.01 ± 5.08	− 4.56 ± 4.17	0.533	*r* = 0.143
Right foot progression angle (°)	Treatment	100	− 2.16 ± 4.97	1.18 ± 4.63	< 0.001	Cohen’s d = 0.692
	Control	19	− 3.15 ± 5.00	− 2.85 ± 4.46	0.433	*r* = 0.185
Walking speed (km/h)	Treatment	100	2.67 ± 0.41	2.55 ± 0.41	0.020	Cohen’s d = − 0.237
	Control	19	2.23 ± 0.43	2.45 ± 0.34	0.113	Cohen’s d = 0.382
Left step length (cm)	Treatment	100	37.90 ± 7.12	41.03 ± 7.62	< 0.001	Cohen’s d = 0.496
	Control	19	31.89 ± 7.43	37.84 ± 7.11	0.002	Cohen’s d = 0.824
Right step length (cm)	Treatment	100	38.42 ± 7.28	41.12 ± 7.64	< 0.001	Cohen’s d = 0.426
	Control	19	31.89 ± 7.99	37.42 ± 7.37	0.005	Cohen’s d = 0.743
Stride length (cm)	Treatment	100	76.26 ± 14.21	82.18 ± 15.04	< 0.001	Cohen’s d = 0.486
	Control	19	63.95 ± 15.18	75.00 ± 14.47	0.003	Cohen’s d = 0.775
Left arch index	Treatment	100	(30.18 ± 7.78)%	(26.65 ± 7.93)%	< 0.001	Cohen’s d = − 0.689
	Control	19	(26.84 ± 7.18)%	(25.96 ± 6.91)%	0.433	Cohen’s d = − 0.184
Right arch index	Treatment	100	(28.95 ± 7.36)%	(26.13 ± 7.35)%	< 0.001	Cohen’s d = − 0.492
	Control	19	(27.95 ± 5.64)%	(27.25 ± 7.31)%	0.536	Cohen’s d = − 0.145
Left forefoot maximum pressure (N/cm^2^)	Treatment	100	15.93 ± 5.92	18.77 ± 6.56	< 0.001	Cohen’s d = 0.639
	Control	19	13.26 ± 5.25	17.18 ± 7.17	< 0.001	Cohen’s d = 1.063
Right forefoot maximum pressure (N/cm^2^)	Treatment	100	15.93 ± 5.92	18.71 ± 6.35	< 0.001	Cohen’s d = 0.666
	Control	19	13.87 ± 4.51	18.35 ± 6.59	< 0.001	Cohen’s d = 1.403
Left midfoot maximum pressure (N/cm^2^)	Treatment	100	7.23 ± 1.55	7.81 ± 2.53	0.009	Cohen’s d = 0.264
	Control	19	7.13 ± 1.75	8.58 ± 2.44	< 0.001	Cohen’s d = 1.008
Right midfoot maximum pressure (N/cm^2^)	Treatment	100	7.36 ± 1.65	8.04 ± 2.45	0.001	Cohen’s d = 0.334
	Control	19	7.02 ± 1.74	8.08 ± 2.00	0.014	Cohen’s d = 0.621
Left heel maximum pressure (N/cm^2^)	Treatment	100	17.88 ± 6.37	21.84 ± 7.13	< 0.001	Cohen’s d = 0.667
	Control	19	19.38 ± 8.42	23.33 ± 8.51	0.004	Cohen’s d = 0.749
Right heel maximum pressure (N/cm^2^)	Treatment	100	17.04 ± 5.58	20.65 ± 5.66	< 0.001	Cohen’s d = 0.754
	Control	19	17.98 ± 6.91	22.26 ± 7.57	0.001	Cohen’s d = 0.896

**Table 3:** Comparison of gait parameters between treatment and control groups at baseline and follow-up

Gait parameters	Testing time	Treatment	Control	*P*-value	Effect sizes
Left foot progression angle (°)	Baseline	− 3.67 ± 4.46	− 5.01 ± 5.08	0.296	Hedges’ g = 0.290
	Follow-up	− 0.24 ± 4.09	− 4.56 ± 4.17	< 0.001	Hedges’ g = 1.046
Right foot progression angle (°)	Baseline	− 2.16 ± 4.97	− 3.15 ± 5.00	0.436	Hedges’ g = 0.197
	Follow-up	1.18 ± 4.63	− 2.85 ± 4.46	< 0.001	*r* = 0.483
Walking speed (km/h)	Baseline	2.67 ± 0.41	2.23 ± 0.43	< 0.001	Hedges’ g = 1.043
	Follow-up	2.55 ± 0.41	2.45 ± 0.34	0.370	*r* = 0.130
Left step length (cm)	Baseline	37.90 ± 7.12	31.89 ± 7.43	0.003	Hedges’ g = 0.832
	Follow-up	41.03 ± 7.62	37.84 ± 7.11	0.088	Hedges’ g = 0.420
Right step length (cm)	Baseline	38.42 ± 7.28	31.89 ± 7.99	0.003	Hedges’ g = 0.877
	Follow-up	41.12 ± 7.64	37.42 ± 7.37	0.057	Hedges’ g = 0.484
Stride length (cm)	Baseline	76.26 ± 14.21	63.95 ± 15.18	0.003	Hedges’ g = 0.852
	Follow-up	82.18 ± 15.04	75.00 ± 14.47	0.060	Hedges’ g = 0.477
Left arch index	Baseline	(30.18 ± 7.78)%	(26.84 ± 7.18)%	0.078	Hedges’ g = 0.431
	Follow-up	(26.65 ± 7.93)%	(25.96 ± 6.91)%	0.699	Hedges’ g = 0.088
Right arch index	Baseline	(28.95 ± 7.36)%	(27.95 ± 5.64)%	0.508	Hedges’ g = 0.139
	Follow-up	(26.13 ± 7.35)%	(27.25 ± 7.31)%	0.547	Hedges’ g = − 0.151
Left forefoot maximum pressure (N/cm^2^)	Baseline	15.93 ± 5.92	13.26 ± 5.25	0.043	*r* = 0.294
	Follow-up	18.77 ± 6.56	17.18 ± 7.17	0.195	*r* = 0.188
Right forefoot maximum pressure (N/cm^2^)	Baseline	15.93 ± 5.92	13.87 ± 4.51	0.211	*r* = 0.182
	Follow-up	18.71 ± 6.35	18.35 ± 6.59	0.957	*r* = 0.008
Left midfoot maximum pressure (N/cm^2^)	Baseline	7.23 ± 1.55	7.13 ± 1.75	0.297	*r* = 0.134
	Follow-up	7.81 ± 2.53	8.58 ± 2.44	0.123	*r* = − 0.214
Right midfoot maximum pressure (N/cm^2^)	Baseline	7.36 ± 1.65	7.02 ± 1.74	0.244	*r* = 0.156
	Follow-up	8.04 ± 2.45	8.08 ± 2.00	0.485	*r* = − 0.094
Left heel maximum pressure (N/cm^2^)	Baseline	17.88 ± 6.37	19.38 ± 8.42	0.509	*r* = − 0.096
	Follow-up	21.84 ± 7.13	23.33 ± 8.51	0.438	*r* = − 0.113
Right heel maximum pressure (N/cm^2^)	Baseline	17.04 ± 5.58	17.98 ± 6.91	0.655	*r* = − 0.065
	Follow-up	20.65 ± 5.66	22.26 ± 7.57	0.446	*r* = − 0.111

See Supplementary Table 2 and 3 for complete values

## Discussion

This study investigated the effects of proprioceptive stimulation foot pads on gait parameters in children with persistent in-toeing gait and demonstrated a significant improvement in FPA following intervention. This finding is consistent with previous research on conservative management strategies for rotational deformities in children. For instance, Nourai et al. [24] reported that long-term conservative management can lead to meaningful improvements in rotational alignment, while studies by Munuera et al. [11]and Sahar Ganjehie et al. [25] further demonstrated that orthotic devices can effectively improve gait angles in pediatric populations. Notably, our results extend this body of evidence by providing quantitative data from a Chinese pediatric cohort—a population underrepresented in the existing literature—thereby supporting the potential applicability of this intervention across diverse clinical settings.

The proposed mechanism of proprioceptive stimulation foot pads is consistent with Bourdiol’s neuromuscular modulation model, in which thin plantar wedges influence muscle tone and postural alignment through altered sensory feedback [19]. Previous studies have shown that even subtle changes in plantar sensory input can affect balance, posture, and gait patterns [26–28]. Investigations by Foisy, Marco, and colleagues demonstrated that thin inserts or heel wedges can improve gait stability and alignment in populations with neurological conditions such as multiple sclerosis and Parkinson’s disease [16, 17]. Park et al. [18] also reported beneficial effects in older adults. The present findings suggest that similar proprioceptive mechanisms may contribute to improved rotational alignment in children with in-toeing gait.

Although FPA improved significantly in the treatment group, other gait parameters—including step length, stride length, arch index, and plantar pressure—showed mixed or trend-level changes without consistent between-group significance. Increases in step length, stride length and plantar pressure were observed in both groups, which likely reflect growth-related factors such as increases in height, body mass, and muscle strength during childhood. This interpretation is consistent with previous reports indicating that spatiotemporal gait variables evolve naturally with maturation [10]. Because both groups experienced comparable developmental changes over the follow-up period, these improvements cannot be attributed solely to the intervention. In addition, measurement variability and increasing familiarity with gait assessment procedures may have contributed to performance changes independent of treatment. This distinction is important for interpreting the limited effects of foot pads on non-rotational gait parameters.

To further contextualize the contribution of maturation, linear gait parameters such as stride length (treatment group: 76.26 ± 14.21 cm to 82.18 ± 15.04 cm, *P* < 0.001, ~ 7.8% increase; control group: 63.95 ± 15.18 cm to 75.00 ± 14.47 cm, *P* = 0.003, ~ 17.3% increase) and peak plantar pressure (e.g., left forefoot: treatment group, 15.93 ± 5.92 to 18.77 ± 6.56 N/cm^2^, *P* < 0.001; control group, 13.26 ± 5.25 to 17.18 ± 7.17 N/cm^2^, *P* < 0.001) were largely influenced by expected developmental gains in leg length (~ 3–6 cm/year) and body weight (~ 3–5 kg/year) among children aged 5–12 years. These effects were comparable between groups, supported by baseline anthropometric equivalence (Table 1; all *P* > 0.05), and therefore do not fully explain the observed between-group difference in non-linear parameters such as FPA (post-intervention *P* < 0.001, effect size = 1.046). Although these findings suggest an association between foot pad use and improvements beyond maturational effects, causal inference is limited by the retrospective study design. Growth normalization (e.g., stride length-to-height ratios) was considered but could not be implemented due to retrospective data constraints; however, baseline comparability and sensitivity analyses partially mitigated this limitation.

The slight reduction in walking speed observed in the treatment group may represent a transitional adaptation associated with improved postural control rather than a decline in functional capacity. Similar compensatory gait adjustments have been reported following sensory-modulating orthotic interventions [10]. Accordingly, reduced walking speed in this context should not be interpreted as functional deterioration.

Despite these targeted biomechanical effects, parental reports indicated high satisfaction with the proprioceptive stimulation foot pads, with perceived reductions in fall frequency and improvements in gait stability. These observations are consistent with findings by Redmond et al. [3], who reported decreased fall rates and increased parental satisfaction following gait plate interventions. Parents also highlighted the convenience of the foot pads and their superior compliance compared with traditional orthotic devices such as the Dennis Brown brace, which is frequently discontinued due to discomfort. As a simple and non-invasive intervention, proprioceptive stimulation foot pads appear to be more readily incorporated into children’s daily activities, supporting their clinical acceptability.

Several limitations of this study should be acknowledged. The retrospective, non-randomized design and the substantial imbalance in group size (treatment *n* = 100 vs. control *n* = 19) may introduce selection bias and reduce statistical precision. Information on adherence to foot pad use was not available, precluding analysis of dose–response relationships. Furthermore, growth-related changes and other unmeasured confounders may have influenced gait parameters over time, and normalization to anthropometric variables could not be performed because of incomplete longitudinal data. Prospective studies with randomized designs, balanced cohorts, adherence monitoring, and growth-normalized outcomes are therefore needed to validate these preliminary findings.

In summary, proprioceptive stimulation foot pads represent a promising, child-friendly conservative intervention for improving FPA in children with in-toeing gait. High parental acceptance further supports their potential real-world utility. By providing novel evidence from a Chinese pediatric cohort, this study contributes to the growing literature on conservative gait correction and supports further investigation into the clinical role of proprioceptive stimulation foot pads in pediatric orthopedic practice.

## Conclusion

In this retrospective cohort study, proprioceptive stimulation foot pads were associated with a significant improvement in FPA, suggesting a potential benefit for correcting rotational alignment in children with in-toeing gait. In contrast, other gait parameters, including step length and stride length, did not demonstrate consistent or statistically significant between-group benefits at follow-up. These findings indicate that the primary effect of this intervention may be limited to rotational gait alignment rather than broader spatiotemporal or plantar pressure outcomes.

Several limitations should be acknowledged. The retrospective, non-randomized design and the substantial imbalance in group size (treatment *n* = 100 vs. control *n* = 19) may introduce selection bias and reduce statistical precision. Information on adherence to foot pad use was unavailable, limiting interpretation of dose–response relationships. In addition, growth-related changes (e.g., height, weight, and motor development) and other unmeasured confounders may have influenced gait parameters over time, and normalization to anthropometric measures could not be performed because of incomplete longitudinal data. Accordingly, these results should be interpreted with caution and considered preliminary.

Future prospective controlled studies with balanced cohorts, adherence monitoring, and growth-normalized outcomes are required to confirm the effectiveness and durability of proprioceptive stimulation foot pads. Further research may also explore their combined use with complementary interventions, such as physical therapy and functional training, to develop more comprehensive and individualized treatment strategies for pediatric gait abnormalities. Mechanistic studies examining neuromuscular control and motor coordination may provide additional insights to refine foot pad design and optimize therapeutic efficiency. Cost-effectiveness analyses and subgroup evaluations—particularly in children with more severe baseline in-toeing—would further clarify their broader clinical applicability.

In summary, as a simple and non-invasive conservative intervention, proprioceptive stimulation foot pads show promise for improving rotational gait alignment in children with in-toeing gait and may represent a feasible option within pediatric orthopedic management.

## Supplementary Information

Below is the link to the electronic supplementary material.

Supplementary Material 1

Supplementary Material 2

Supplementary Material 3

Supplementary Material 4

Supplementary Material 5

## Data Availability

All data and research materials will be made available upon request, in accordance with privacy and ethical regulations.
