# Supplementary material for: Effects of proprioceptive stimulation foot pads on in-toeing gait in children: a retrospective study
Source: J Orthop Surg Res. 2026 Feb 1;21:168. doi: 10.1186/s13018-025-06644-9 (PMC12951979; doi:10.1186/s13018-025-06644-9)
Supplement: Supplementary file 1 — Supplementary Material 1 [file 13018_2025_6644_MOESM1_ESM.docx]

Fig.1 Original Data Display of Various Gait Parameters: A: Foot Scan

It displays the plantar scans of both feet, focusing on the overall shape of the feet and the pressure areas on the soles. Plantar Regions:The heel and forefoot areas show different levels of pressure, with darker colors representing greater pressure in those regions. This helps in assessing the arch height and its corresponding functional state.

B: Gait Parameters

It illustrates various gait parameters, including loading response, single limb support, pre-swing, and swing phases. Gait Phases:The different phases of the gait cycle are marked, from initial contact to swing and back to contact, providing an understanding of the sequence in the gait process. Geometric Gait Data:Includes foot progression angle, step length, stride length, and step width. Each parameter is measured separately for the left and right foot and is presented in bar charts with mean values and standard deviations, allowing for an assessment of gait symmetry and trends.

C: Time Parameters

It shows a comparison of different temporal parameters during the gait cycle. Step and Stride Time:This section includes step time, stride time, cadence, and speed. The differences between the left and right foot step times are highlighted with different colors, helping to evaluate gait rhythm and consistency. Cadence and Speed:Cadence is shown in steps per minute, while speed is displayed in kilometers per hour. Changes across groups are visually compared using bar charts, aiding in understanding speed changes post-intervention.

D: Forefoot to Rearfoot Pressure Distribution Ratio

It shows the pressure distribution between the forefoot and the rearfoot. Pressure Plot:

The 3D plots illustrate the pressure across different regions of the sole, with deeper colors representing higher pressure, showing the weight distribution between the forefoot and the rearfoot. Pressure Ratio Bar Chart:The pressure ratio between different regions is shown using bar charts, which helps in understanding the distribution of pressure between the forefoot and rearfoot during gait.

E: Center of Pressure Analysis - Butterfly Shape

It shows the trajectory of the center of pressure for the left and right feet, forming a "butterfly shape" pattern. Butterfly Trajectory:The lines in different colors indicate the movement trajectory of the center of pressure over time, displaying gait stability and symmetry. These trajectories are useful for assessing balance during gait and control effectiveness.

F: Gait Line

It displays the gait lines for the left and right feet.Gait Trajectory:The gait lines represent the contact path of the sole during each step. By analyzing the shape and symmetry of these gait lines, one can evaluate gait stability and control efficiency.

G: Tri-Zone Pressure Analysis

It presents the pressure distribution across different areas of the foot (heel, midfoot, and forefoot).Maximum Force in Each Zone:The maximum pressure in each zone is represented by heat maps, with deeper colors indicating greater pressure. This helps identify which area of the foot bears the most load, pointing to potential gait imbalance issues.

H: Maximum Pressure (N/cm²)

It shows the maximum pressure values for the different foot regions using bar charts.Peak Pressure Comparison:Maximum pressure values for the left and right foot in the forefoot, midfoot, and heel areas are measured, with values presented as mean ± standard deviation. This aids in visually comparing the pressure differences across different regions.
