# Supplementary material for: Effects of proprioceptive stimulation foot pads on in-toeing gait in children: a retrospective study
Source: J Orthop Surg Res. 2026 Feb 1;21:168. doi: 10.1186/s13018-025-06644-9 (PMC12951979; doi:10.1186/s13018-025-06644-9)
Supplement: Supplementary file 2 — Supplementary Material 2 [file 13018_2025_6644_MOESM2_ESM.pdf]

zebris
步态分析与训练系统

zebris 步态分析训练系统集成了先进的足底压力分布测量技术，可以准确快速的进行临床步态分析评估和生物反馈模式的步态训练。系统主体的医用慢速跑台的跑带下方内置了上千个高精度标准化的电容式压力分布传感器，能测量和分析步行时的动态足底压力分布，数据通过 USB 接口直接传输至 PC 电脑端。

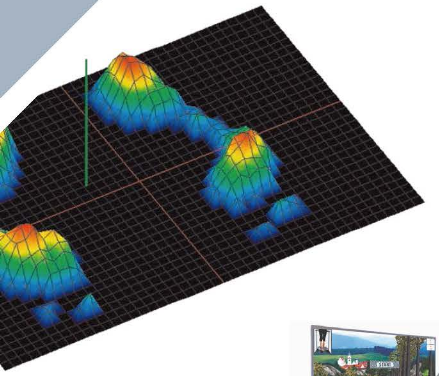

足底压力分布测量平板

平板内置电容式压力传感器，可以快速准确地进行足底压力分析，支持步态分析和静态平衡测试软件自带步态分析报告，包括：步长、步宽、步速、步向角、步态中心线、力变化曲线等步态参数。

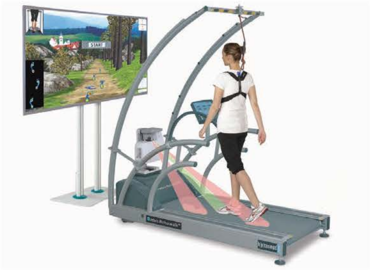

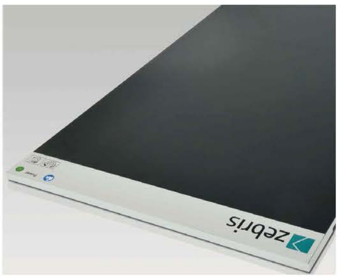

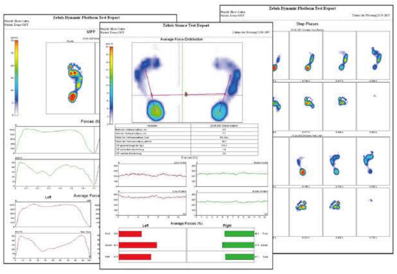

型号参数

型号	平板尺寸	传感器面积	传感器数量	采样频率	PC 端接口
FDM SX	500x400x21mm	400x300mm	1920	120Hz	USB
FDM S	690x400x21mm	540x330mm	2560	120Hz<br>可选 240Hz	
FDM1.5	1580x650x21mm	1490x542mm	11264	100Hz<br>可选<br>200/200Hz	
FDM 2	2120x650x21mm	2030x542mm	15360	100Hz<br>可选 200Hz	
FDM 3	3070x605x21mm	2980x542mm	22528	100Hz	
PDM-XS	570x400x15mm	406x339mm	1920	200Hz	USB
PDM-XS mobile					USB/ 蓝牙
PDM-S	710x400x15mm	542x339mm	2560	200Hz	USB
PDM-S mobile					USB/ 蓝牙
PDM-L	1370x535x15mm	1220x474mm	8064	120Hz	USB
PDM-L mobile					USB/ 蓝牙

* 支持拼接使用：2xFDM1.5，2xFDM 2 或 2xFDM 3

步态分析及训练跑台

测试功能

集成压力分布测量系统

快速、准确地完成临床步态分析

训练功能

投影训练模块，个性化订制步态训练方案

情景互动模式，增强训练的趣味

特点

- 跑台的履带内置电容式压力传感器
- 多种型号，可选不同的尺寸和配速，满足不同测试需求
- 软件报告提供：步长、步宽、步速、步向角、步行时相、步态中心线、力变化曲线等步态参数
- 可与表面肌电、二维视频同步测试

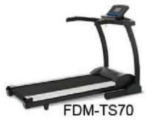
FDM-TS70

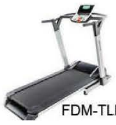
FDM-TLK3

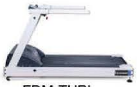
FDM-THPL

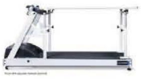
FDM-THM

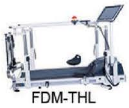
FDM-THL

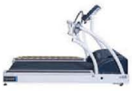
FDM-THQ

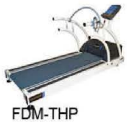
FDM-THP

balori 协调性训练系统

功能

利用 zebris 压力分布测量平板、拍摄身体运动的摄像机和虚拟现实训练软件，提供个性化的全身平衡和协调性训练适应于健身训练、临床康复治疗和老年人平衡功能训练 。

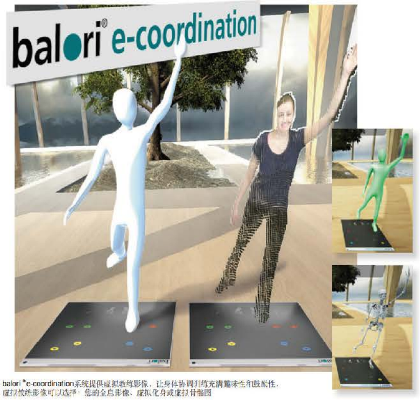

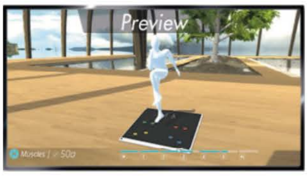

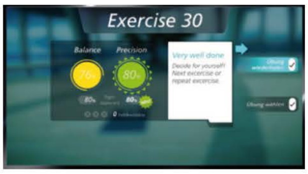

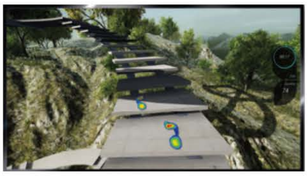

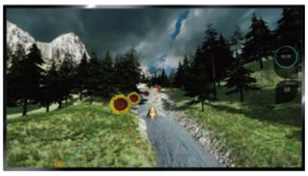

特点

符合日常生活活动的特点。

提高神经肌肉系统的反应速度防跌倒的平衡训练，可以提高老年人对环境的适应性核心力量训练，让您的腰部和背部更健康。

软件界面直观，有客观的训练参数，通过虚拟的动画形象实现简单的虚拟三维教学。

配置方案

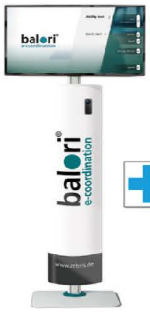

- 一体立式屏幕
- 高性能 PC 主机
- 3D 摄像机记录运动
- 配套 balori® 软件
- 提供虚拟互动
- 身体协调性训练模块

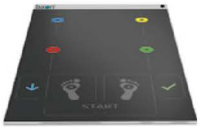

- zebris 压力分布测量平板
  型号：balori®-X
  规格尺寸：115 x 60 x 2 cm
  传感器数量：1456 个

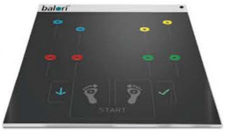

- zebris 压力分布测量平板
  型号：balori®-C
  规格尺寸：122 x 90 x 2 cm
  传感器数量：2240 个
