# Supplementary material for: Effects of proprioceptive stimulation foot pads on in-toeing gait in children: a retrospective study
Source: J Orthop Surg Res. 2026 Feb 1;21:168. doi: 10.1186/s13018-025-06644-9 (PMC12951979; doi:10.1186/s13018-025-06644-9)
Supplement: Supplementary file 3 — Supplementary Material 3 [file 13018_2025_6644_MOESM3_ESM.pdf]

The zebris FDM-T System for stance and gait analysis

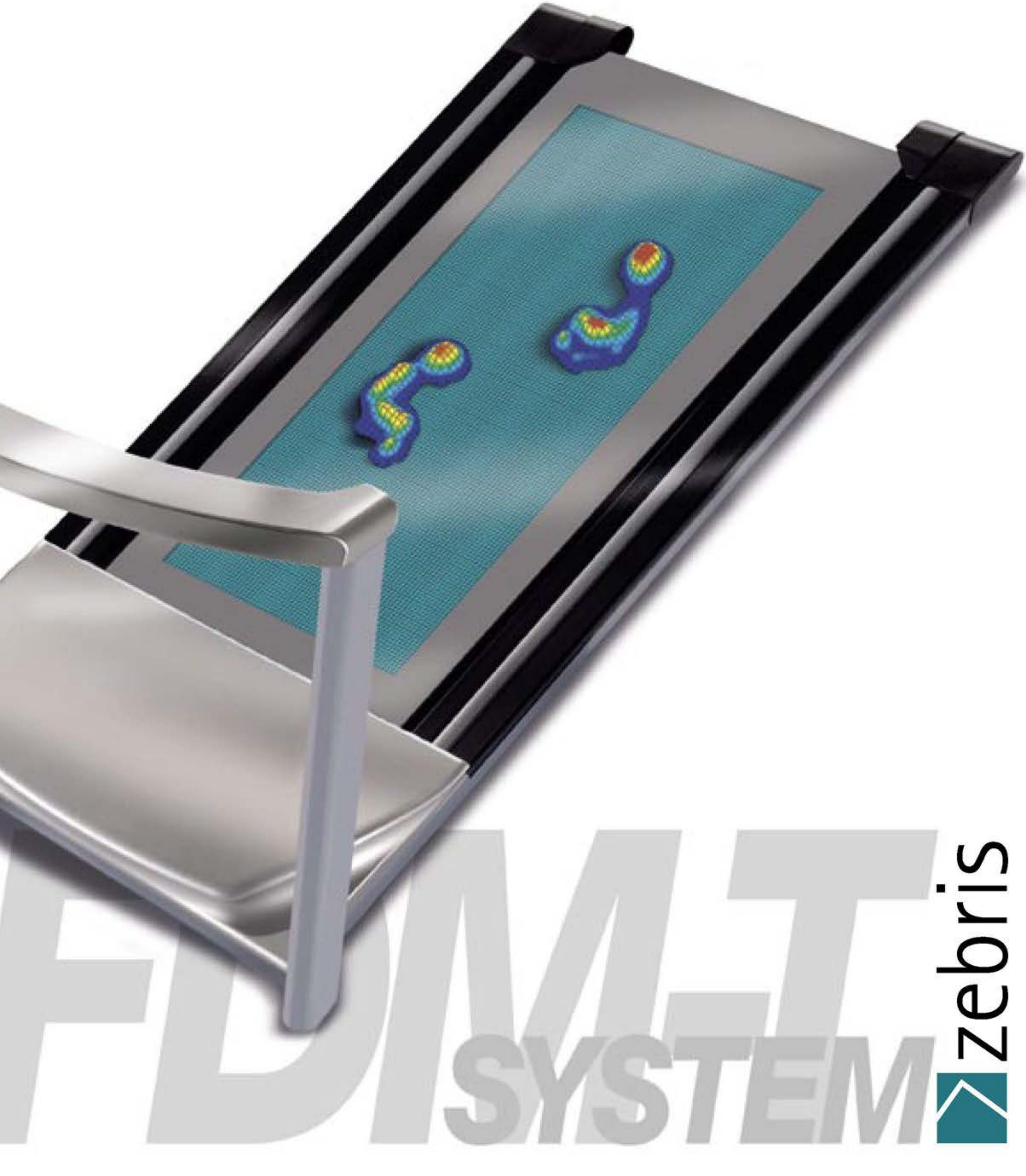

zebris

The zebris FDM-T System - a stance and g

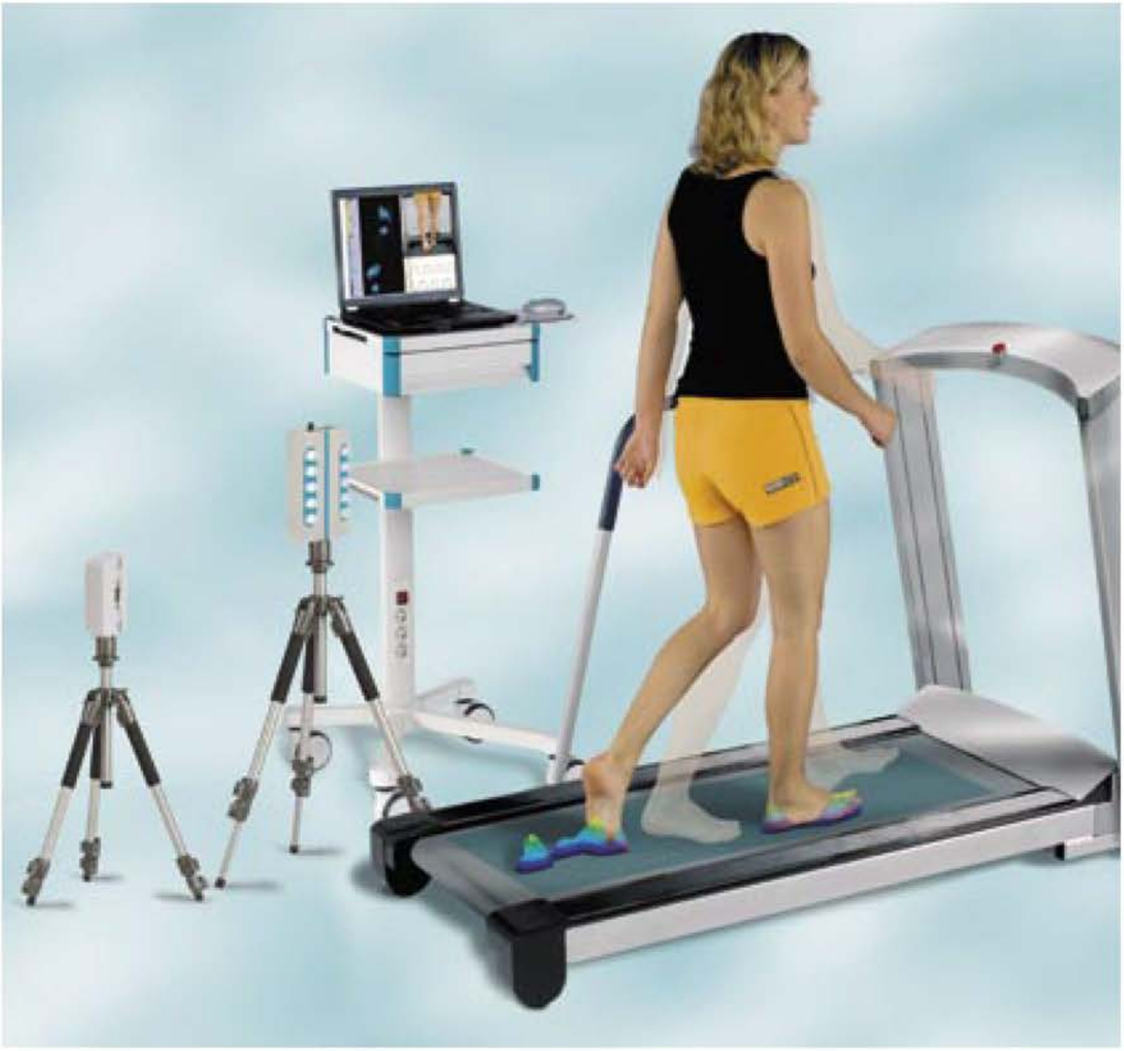

Using the zebris FDM-T Analysis Center, gait and roll-off analyses can be carried out easily and quickly.
The basic system can be extended in a variety of ways with video, motion analysis and EMG.

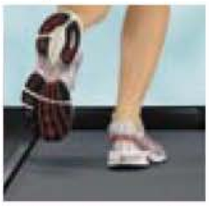

The treadmill can be used with shoes on or bare-foot. As a result, the influence of the shoes on the roll-off behavior can be examined.

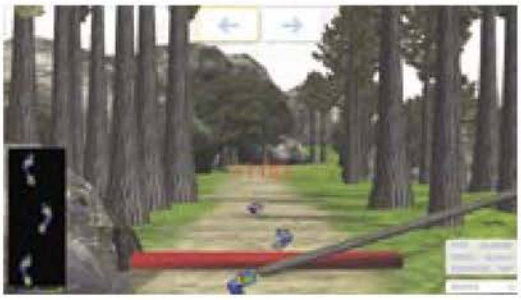

The evaluation software enables gait training to be carried out in a virtual running environment. Whilst watching his own footprints, the runner performs certain tasks which demand a constant variation in his walking and balancing mode.

The system consists of a treadmill ergometer with an integrated, calibrated measuring sensor matrix. This consists of numerous high-quality capacitive force sensors. Using a system specially developed by zebris the movement of the treadmill is compensated so that completely stable gait and roll-off patterns can be analyzed. Different types of treadmill ergometer are available for the basic system.

For the stance analysis, the force distribution and the posture are recorded and evaluated.

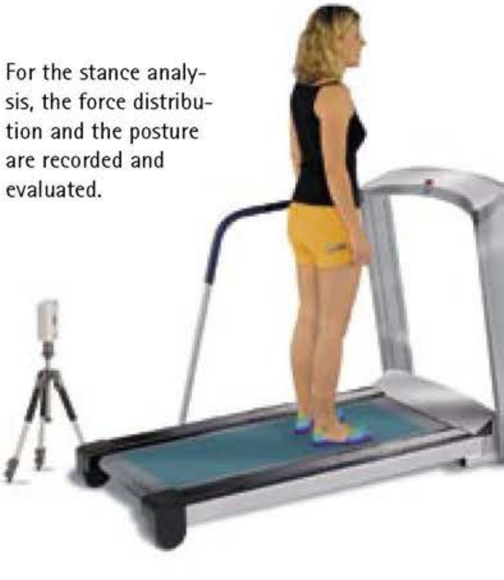

ait analysis center

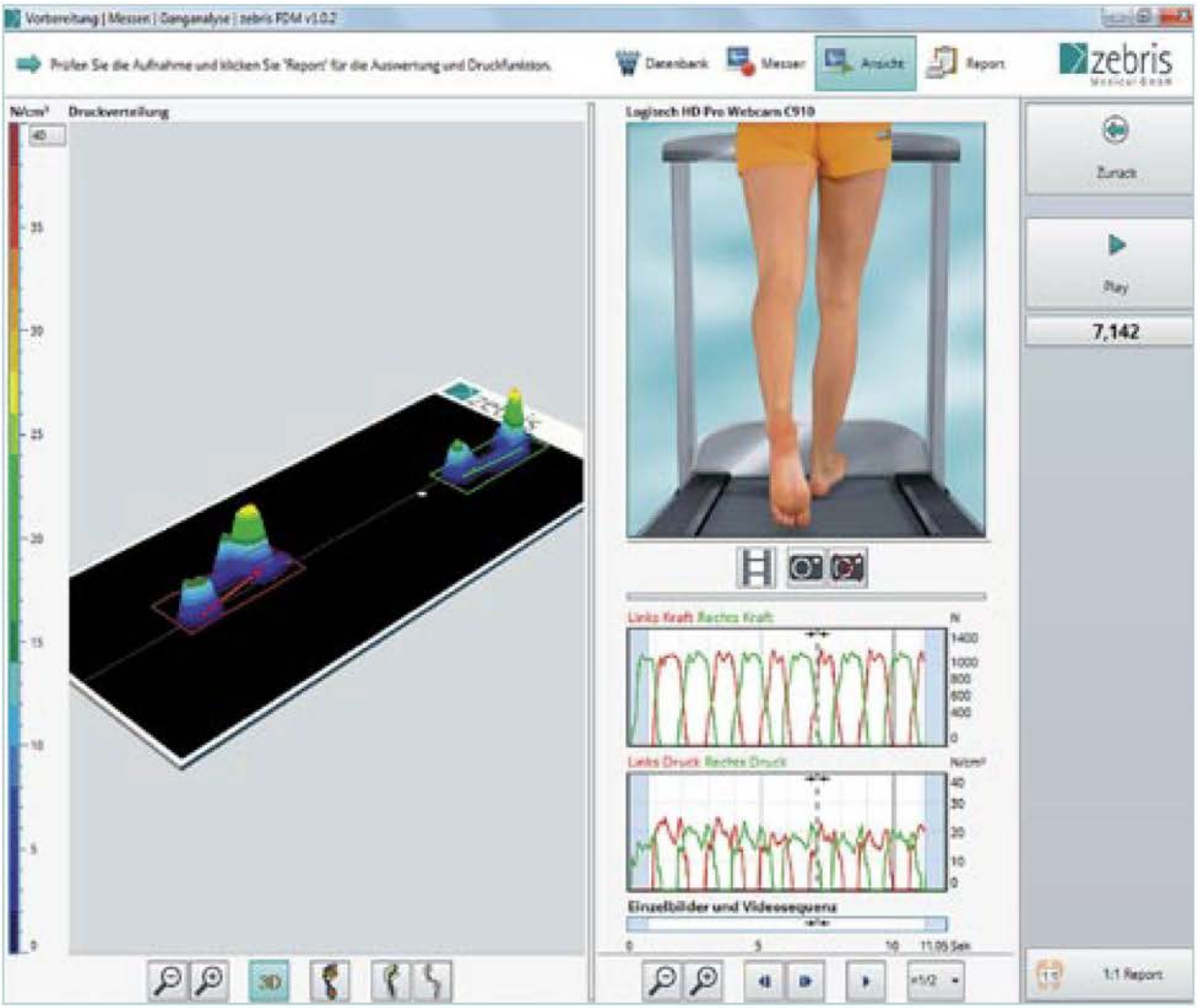

The display of all the measuring results is effected in real-time using commercially available PCs.
A feedback control of the force on the extremities is thereby possible.
The software provides a „Signal Viewer" in which all the recorded measuring variables are displayed in slow motion and completely synchronized.
The allocation of the floor contacts to the left or right side of the body is carried out fully automatically by the evaluation software.

With one mouse click the measuring results are shown on the computer and can be printed out in color in the Report. This Report is set up over several pages and includes a table with important position and time parameters, such as the stance, swing and double-standing phases, step-lengths, etc., with a direct comparison of both sides.

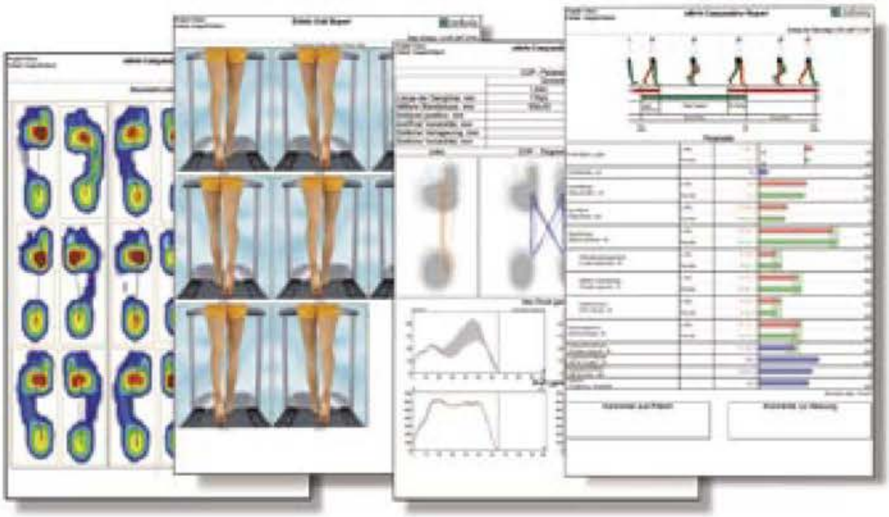

On other pages in the Report, the lines of the centers of pressure (COP) are automatically measured.
The force curves are divided into the left and right side of the body, averaged and normalized to 100% of the step cycle.
Selected roll-off procedures are displayed in the Viewer as color-coded force distribution images (MPP).

zebris

The zebris FDM-T System with numerous possibilities for extension

The basic FDM-T measuring system consists of the instrumented treadmill ergometer with instruments and the PC linked via a USB interface. Depending on the design, the sensor unit is fitted with up to 12,000 pressure sensors.

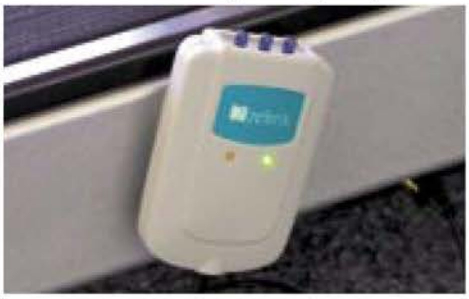

All the FDM-T systems are equiped in their standard form with a video synchronization output for time synchronization with the camera.

The time synchronization using the optionally available cordless radio adapter DAB is effected via an infrared interface. The radio adapter is connected to the PC via a Bluetooth interface and can be fitted with up to eight EMG amplifiers.

Depending on the design, the treadmill ergometer can have an additional input and output that enables any other external devices to be synchronized. Where implementation for rehabilitation purposes is concerned, an optional projector is available for the visual stimulation.

The infrared synchronization adapter is connected to the junction box of the treadmill ergometer.

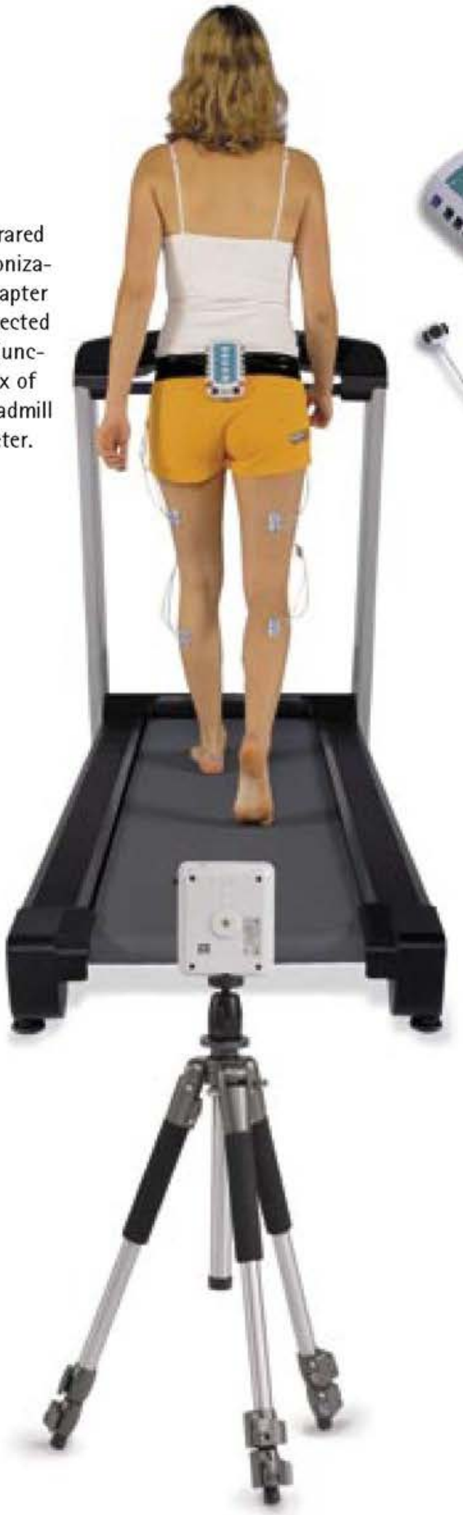

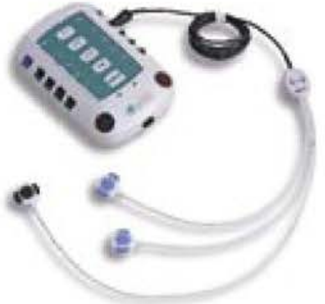

Besides the infrared interface, the cordless radio adapter has eight analog inputs, four digital inputs and an output for directly connecting a special USB adapter cable.

In addition to the camera and tripod, the SYNCCam camera module includes all the connection and synchronization cables necessary for operating it, as well as the software enhancements.

For creating optimum lighting conditions, the synchronized illumination systems, SYNCLight and SYNCLight plus, are available.

©zebris 9/2012.
Subject to technical modifications.

zebris Medical GmbH · Germany · Postfach 1143 · D-88305 Isny im Allgäu · Max-Eyth-Weg 43 · D-88316 Isny im Allgäu
Telefon +49-7562-97260 · Telefax +49-7562-972650 · info@zebris.de · www.zebris.de
