# Supplementary material for: Effects of proprioceptive stimulation foot pads on in-toeing gait in children: a retrospective study
Source: J Orthop Surg Res. 2026 Feb 1;21:168. doi: 10.1186/s13018-025-06644-9 (PMC12951979; doi:10.1186/s13018-025-06644-9)
Supplement: Supplementary file 5 — Supplementary Material 5 [file 13018_2025_6644_MOESM5_ESM.docx]

**Table 2: Within-Group Changes in Gait Parameters at Baseline and Follow-up**

| **Gait Parameters** | **Group** | ***n*** | **Baseline** | **Follow-up** | **Difference & 95% CI**  **(F-B)** | ***Statistical test*** | ***P-value*** | ***effect sizes*** |
| --- | --- | --- | --- | --- | --- | --- | --- | --- |
| Left Foot Progression Angle(°) | Treatment | 100 | -3.67±4.46 | -0.24±4.09 | 3.43  (2.64 to 4.22) | Paired t-test | <0.001 | Cohen’s d = 0.861 |
|  | Control | 19 | -5.01±5.08 | -4.56±4.17 | 0.45  (-1.17 to 2.06) | Wilcoxon signed-rank | 0.533 | r = 0.143 |
| Right Foot Progression Angle(°) | Treatment | 100 | -2.16±4.97 | 1.18±4.63 | 3.34  (2.38 to 4.30) | Paired t-test | <0.001 | Cohen’s d = 0.692 |
|  | Control | 19 | -3.15±5.00 | -2.85±4.46 | 0.29  (-1.05 to 1.64) | Wilcoxon signed-rank | 0.433 | r = 0.185 |
| Walking Speed(km/h) | Treatment | 100 | 2.67±0.41 | 2.55±0.41 | -0.12  (-0.21 to -0.02) | Paired t-test | 0.020 | Cohen’s d = -0.237 |
|  | Control | 19 | 2.23±0.43 | 2.45±0.34 | 0.22  (0.06 to 0.49) | Paired t-test | 0.113 | Cohen’s d = 0.382 |
| Left Step Length(cm) | Treatment | 100 | 37.90±7.12 | 41.03±7.62 | 3.13  (1.88 to 4.38) | Paired t-test | <0.001 | Cohen’s d = 0.496 |
|  | Control | 19 | 31.89±7.43 | 37.84±7.11 | 5.95  (2.47 to 9.43) | Paired t-test | 0.002 | Cohen’s d = 0.824 |
| Right Step Length(cm) | Treatment | 100 | 38.42±7.28 | 41.12±7.64 | 2.70  (1.44 to 3.96) | Paired t-test | <0.001 | Cohen’s d = 0.426 |
|  | Control | 19 | 31.89±7.99 | 37.42±7.37 | 5.53  (1.94 to 9.11) | Paired t-test | 0.005 | Cohen’s d = 0.743 |
| Stride Length(cm) | Treatment | 100 | 76.26±14.21 | 82.18±15.04 | 5.92  (3.50 to 8.34) | Paired t-test | <0.001 | Cohen’s d = 0.486 |
|  | Control | 19 | 63.95±15.18 | 75.00±14.47 | 11.05  (4.18 to 17.92) | Paired t-test | 0.003 | Cohen’s d = 0.775 |
| Left Arch Index | Treatment | 100 | (30.18±7.78)% | (26.65±7.93)% | -3.53%  (-4.54 to -2.51)% | Paired t-test | <0.001 | Cohen’s d = -0.689 |
|  | Control | 19 | (26.84±7.18)% | (25.96±6.91)% | -0.88%  (-3.17 to 1.14)% | Paired t-test | 0.433 | Cohen’s d = -0.184 |
| Right Arch Index | Treatment | 100 | (28.95±7.36)% | (26.13±7.35)% | -2.81%  (-3.95 to -1.68)% | Paired t-test | <0.001 | Cohen’s d = -0.492 |
|  | Control | 19 | (27.95±5.64)% | (27.25±7.31)% | -0.70%  (-3.03 to 1.63)% | Paired t-test | 0.536 | Cohen’s d = -0.145 |
| Left Forefoot Maximum Pressure(N/cm^2^) | Treatment | 100 | 15.93±5.92 | 18.77±6.56 | 2.84  (1.96 to 3.72) | Paired t-test | <0.001 | Cohen’s d = 0.639 |
|  | Control | 19 | 13.26±5.25 | 17.18±7.17 | 3.92  (2.14 to 5.70) | Paired t-test | <0.001 | Cohen’s d = 1.063 |
| Right Forefoot Maximum Pressure(N/cm^2^) | Treatment | 100 | 15.93±5.92 | 18.71±6.35 | 2.76  (1.95 to 3.60) | Paired t-test | <0.001 | Cohen’s d = 0.666 |
|  | Control | 19 | 13.87±4.51 | 18.35±6.59 | 4.48  (2.94 to 6.02) | Paired t-test | <0.001 | Cohen’s d = 1.403 |
| Left Midfoot Maximum Pressure(N/cm^2^) | Treatment | 100 | 7.23±1.55 | 7.81±2.53 | 0.58  (0.15 to 1.01) | Paired t-test | 0.009 | Cohen’s d = 0.264 |
|  | Control | 19 | 7.13±1.75 | 8.58±2.44 | 1.45  (0.77 to 2.12) | Paired t-test | <0.001 | Cohen’s d = 1.008 |
| Right Midfoot Maximum Pressure(N/cm^2^) | Treatment | 100 | 7.36±1.65 | 8.04±2.45 | 0.68  (0.28 to 1.08) | Paired t-test | 0.001 | Cohen’s d = 0.334 |
|  | Control | 19 | 7.02±1.74 | 8.08±2.00 | 1.06  (0.24 to 1.89) | Paired t-test | 0.014 | Cohen’s d = 0.621 |
| Left Heel Maximum Pressure(N/cm^2^) | Treatment | 100 | 17.88±6.37 | 21.84±7.13 | 3.96  (2.78 to 5.14) | Paired t-test | <0.001 | Cohen’s d = 0.667 |
|  | Control | 19 | 19.38±8.42 | 23.33±8.51 | 3.95  (1.41 to 6.50) | Paired t-test | 0.004 | Cohen’s d = 0.749 |
| Right Heel Maximum Pressure(N/cm^2^) | Treatment | 100 | 17.04±5.58 | 20.65±5.66 | 3.61  (2.66 to 4.56) | Paired t-test | <0.001 | Cohen’s d = 0.754 |
|  | Control | 19 | 17.98±6.91 | 22.26±7.57 | 4.28  (1.94 to 6.62) | Paired t-test | 0.001 | Cohen’s d = 0.896 |

**Table 3: Comparison of Gait Parameters Between Treatment and Control Groups at Baseline and Follow-up**

| **Gait Parameters** | **Testing Time** | **Treatment** | **Control** | **Difference & 95% CI**  **（T-C）** | ***Statistical test*** | ***P-value*** | ***effect sizes*** |
| --- | --- | --- | --- | --- | --- | --- | --- |
| Left Foot Progression Angle(°) | Baseline | -3.67±4.46 | -5.01±5.08 | 1.33  (-0.93 to 3.59) | Welch’s t-test | 0.296 | Hedges’ g = 0.290 |
|  | Follow-up | -0.24±4.09 | -4.56±4.17 | 4.32  (2.28 to 6.35) | Welch’s t-test | <0.001 | Hedges’ g = 1.046 |
| Right Foot Progression Angle(°) | Baseline | -2.16±4.97 | -3.15±5.00 | 0.99  (-1.48 to 3.45) | Welch’s t-test | 0.436 | Hedges’ g = 0.197 |
|  | Follow-up | 1.18±4.63 | -2.85±4.46 | 4.03  (1.75 to 6.32) | Mann–Whitney U | <0.001 | r = 0.483 |
| Walking Speed(km/h) | Baseline | 2.67±0.41 | 2.23±0.43 | 0.44  (0.23 to 0.64) | Welch’s t-test | <0.001 | Hedges’ g = 1.043 |
|  | Follow-up | 2.55±0.41 | 2.45±0.34 | 0.10  (-0.09 to 0.30) | Mann–Whitney U | 0.370 | r = 0.130 |
| Left Step Length(cm) | Baseline | 37.90±7.12 | 31.89±7.43 | 6.00  (2.45 to 9.56) | Welch’s t-test | 0.003 | Hedges’ g = 0.832 |
|  | Follow-up | 41.03±7.62 | 37.84±7.11 | 3.19  (-0.55 to 6.93) | Welch’s t-test | 0.088 | Hedges’ g = 0.420 |
| Right Step Length(cm) | Baseline | 38.42±7.28 | 31.89±7.99 | 6.53  (2.86 to 10.19) | Welch’s t-test | 0.003 | Hedges’ g = 0.877 |
|  | Follow-up | 41.12±7.64 | 37.42±7.37 | 3.70  (-0.07 to 7.46) | Welch’s t-test | 0.057 | Hedges’ g = 0.484 |
| Stride Length(cm) | Baseline | 76.26±14.21 | 63.95±15.18 | 12.31  (5.19 to 19.43) | Welch’s t-test | 0.003 | Hedges’ g = 0.852 |
|  | Follow-up | 82.18±15.04 | 75.00±14.47 | 7.18  (-0.31 to 14.67) | Welch’s t-test | 0.060 | Hedges’ g = 0.477 |
| Left Arch Index | Baseline | (30.18±7.78)% | (26.84±7.18)% | 3.34%  (-0.47 to 7.16)% | Welch’s t-test | 0.078 | Hedges’ g = 0.431 |
|  | Follow-up | (26.65±7.93)% | (25.96±6.91)% | 0.69%  (-3.16 to 4.55)% | Welch’s t-test | 0.699 | Hedges’ g = 0.088 |
| Right Arch Index | Baseline | (28.95±7.36)% | (27.95±5.64)% | 1.00%  (-2.54 to 4.53)% | Welch’s t-test | 0.508 | Hedges’ g = 0.139 |
|  | Follow-up | (26.13±7.35)% | (27.25±7.31)% | -1.12%  (-4.76 to 2.52)% | Welch’s t-test | 0.547 | Hedges’ g = -0.151 |
| Left Forefoot Maximum Pressure(N/cm^2^) | Baseline | 15.93±5.92 | 13.26±5.25 | 2.67  (-0.22 to 5.55) | Mann–Whitney U | 0.043 | r = 0.294 |
|  | Follow-up | 18.77±6.56 | 17.18±7.17 | 1.58  (-1.72 to 4.88) | Mann–Whitney U | 0.195 | r = 0.188 |
| Right Forefoot Maximum Pressure(N/cm^2^) | Baseline | 15.93±5.92 | 13.87±4.51 | 2.07  (-0.77 to 4.90) | Mann–Whitney U | 0.211 | r = 0.182 |
|  | Follow-up | 18.71±6.35 | 18.35±6.59 | 0.36  (-2.81 to 3.53) | Mann–Whitney U | 0.957 | r = 0.008 |
| Left Midfoot Maximum Pressure(N/cm^2^) | Baseline | 7.23±1.55 | 7.13±1.75 | 0.09  (-0.69 to 0.88) | Mann–Whitney U | 0.297 | r = 0.134 |
|  | Follow-up | 7.81±2.53 | 8.58±2.44 | -0.77  (-2.02 to 0.47) | Mann–Whitney U | 0.123 | r = -0.214 |
| Right Midfoot Maximum Pressure(N/cm^2^) | Baseline | 7.36±1.65 | 7.02±1.74 | 0.34  (-0.48 to 1.16) | Mann–Whitney U | 0.244 | r = 0.156 |
|  | Follow-up | 8.04±2.45 | 8.08±2.00 | -0.04  (-1.23 to 1.14) | Mann–Whitney U | 0.485 | r = -0.094 |
| Left Heel Maximum Pressure(N/cm^2^) | Baseline | 17.88±6.37 | 19.38±8.42 | -1.50  (-4.83 to 1.83) | Mann–Whitney U | 0.509 | r = -0.096 |
|  | Follow-up | 21.84±7.13 | 23.33±8.51 | -1.49  (-5.14 to 2.16) | Mann–Whitney U | 0.438 | r = -0.113 |
| Right Heel Maximum Pressure(N/cm^2^) | Baseline | 17.04±5.58 | 17.98±6.91 | -0.94  (-3.81 to 1.94) | Mann–Whitney U | 0.655 | r = -0.065 |
|  | Follow-up | 20.65±5.66 | 22.26±7.57 | -1.61  (-4.58 to 1.36) | Mann–Whitney U | 0.446 | r = -0.111 |

Notes:

Data are presented as mean ± standard deviation. Statistical significance was assessed using two-sided p values, with p < 0.05 considered statistically significant. Effect sizes were reported to quantify the magnitude of change or difference and were interpreted according to conventional thresholds: small (≈ 0.2), medium (≈ 0.5), and large (≥ 0.8).

Table 2 reports within-group comparisons between baseline and follow-up for the treatment and control groups separately. Paired t-tests were used for normally distributed variables, and Wilcoxon signed-rank tests were applied for non-normally distributed variables. Although the control group sample size was relatively small, t-tests were considered appropriate when distributional assumptions were reasonably satisfied; nevertheless, the results should be interpreted with caution, and effect sizes were reported to aid interpretation beyond statistical significance. Differences were calculated as follow-up minus baseline (F-B). Cohen’s d was reported for parametric tests, and the effect size r was reported for non-parametric tests.

Table 3 presents between-group comparisons between the treatment and control groups at baseline and follow-up. Welch’s t-tests were used for normally distributed variables to account for unequal variances and sample sizes, and Mann–Whitney U tests were applied for non-normally distributed variables. Differences were calculated as treatment minus control (T-C). Effect sizes were reported as Hedges’ g for parametric tests, incorporating a small-sample correction due to unequal group sizes, and as the effect size r for non-parametric tests.
